# Heterogeneity in the effect of marked weight loss on metabolic function in women with obesity

**DOI:** 10.1172/jci.insight.169541

**Published:** 2023-06-22

**Authors:** Bettina Mittendorfer, Brandon D. Kayser, Mihoko Yoshino, Jun Yoshino, Jeramie D. Watrous, Mohit Jain, J. Christopher Eagon, Bruce W. Patterson, Samuel Klein

**Affiliations:** 1 Center for Human Nutrition, Washington University School of Medicine, St. Louis, Missouri, USA.; 2 Genentech, South San Francisco, California, USA.; 3 Department of Medicine, UCSD, La Jolla, California, USA.; 4 Sansum Diabetes Research Institute, Santa Barbara, California, USA.

**Keywords:** Metabolism, Obesity

## Abstract

**BACKGROUND:**

There is considerable heterogeneity in the effect of weight loss on metabolic function in people with obesity.

**METHODS:**

We evaluated muscle and liver insulin sensitivity, body composition, and circulating factors associated with insulin action before and after approximately 20% weight loss in women identified as “Responders” (*n* = 11) or “Non-responders” (*n* = 11), defined as the top (>75% increase) and bottom (<5% increase) quartiles of the weight loss–induced increase in glucose disposal rate (GDR) during a hyperinsulinemic-euglycemic clamp procedure, among 43 women with obesity (BMI: 44.1 ± 7.9 kg/m^2^).

**RESULTS:**

At baseline, GDR, which provides an index of muscle insulin sensitivity, and the hepatic insulin sensitivity index were more than 50% lower in Responders than Non-responders, but both increased much more after weight loss in Responders than Non-responders, which eliminated the differences between groups. Weight loss also caused greater decreases in intrahepatic triglyceride content and plasma adiponectin and PAI-1 concentrations in Responders than Non-responders and greater insulin-mediated suppression of plasma free fatty acids, branched-chain amino acids, and C3/C5 acylcarnitines in Non-responders than Responders, so that differences between groups at baseline were no longer present after weight loss. The effect of weight loss on total body fat mass, intra-abdominal adipose tissue volume, adipocyte size, and circulating inflammatory markers were not different between groups.

**CONCLUSION:**

The results from our study demonstrate that the heterogeneity in the effects of marked weight loss on muscle and hepatic insulin sensitivity in people with obesity is determined by baseline insulin action, and reaches a ceiling when “normal” insulin action is achieved.

**TRIAL REGISTRATION:**

NCT00981500, NCT01299519, NCT02207777.

**FUNDING:**

NIH grants P30 DK056341, P30 DK020579, P30 DK052574, UL1 TR002345, and T32 HL13035, the American Diabetes Association (1-18-ICTS-119), the Longer Life Foundation (2019-011), and the Atkins Philanthropic Trust.

## Graphical abstract

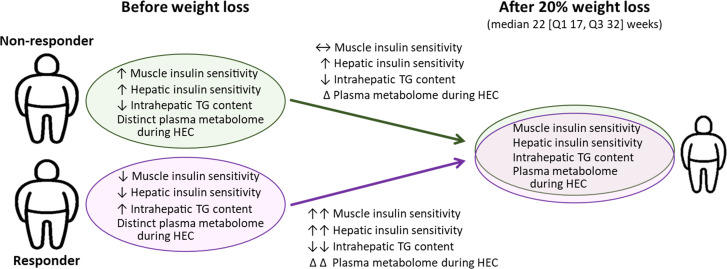

## Introduction

Insulin-resistant glucose metabolism is the most common metabolic complication associated with obesity and is an important risk factor for developing type 2 diabetes and other cardiometabolic diseases, such as nonalcoholic fatty liver disease and atherosclerosis (1–4). Weight loss improves multiorgan insulin sensitivity (5–7) and reduces the risk of developing type 2 diabetes (8–10). However, in reviewing the data from our previous (6, 11, 12) and ongoing studies, we found considerable heterogeneity in the effect of weight loss on muscle insulin sensitivity, assessed as insulin-stimulated glucose disposal rate in relation to fat-free mass (FFM) during a hyperinsulinemic-euglycemic clamp procedure (13). In fact, some participants had no improvement in muscle insulin sensitivity at all, despite marked (>15%) weight loss. The phenotypic and physiological features that distinguish “metabolic Responders” and “metabolic Non-responders” to marked weight loss are not known. It is possible that the heterogeneity in the metabolic response is related to the heterogeneity of baseline metabolic function among people with obesity (1).

The purpose of the present study was to evaluate baseline muscle and liver insulin sensitivity and metabolic factors associated with insulin resistance (i.e., percentage body mass as fat, intra-abdominal adipose tissue volume, intrahepatic triglyceride content, adipocyte size, and specific circulating hormone, inflammatory protein, and metabolite concentrations) and the weight loss–induced changes in these metabolic variables in women with obesity who were considered metabolic Responders or Non-responders to marked (16%–25%) weight loss. Responders and Non-responders were identified as participants within the top (>75% increase) and bottom (<5% increase) quartiles of the increase in insulin-stimulated glucose disposal rate during a hyperinsulinemic-euglycemic clamp procedure among 43 women with class II and class III obesity (body mass index ≥35 kg/m^2^). We hypothesized that (i) Non-responders would be more insulin sensitive than Responders at baseline and metabolic factors associated with insulin resistance would be attenuated in Non-responders compared with Responders at baseline, and (ii) weight loss would cause a greater change in multiorgan insulin sensitivity and metabolic factors associated with insulin resistance in Responders than Non-responders.

## Results

### Glucose kinetics and insulin sensitivity

#### Skeletal muscle insulin sensitivity.

**Figure 1:**
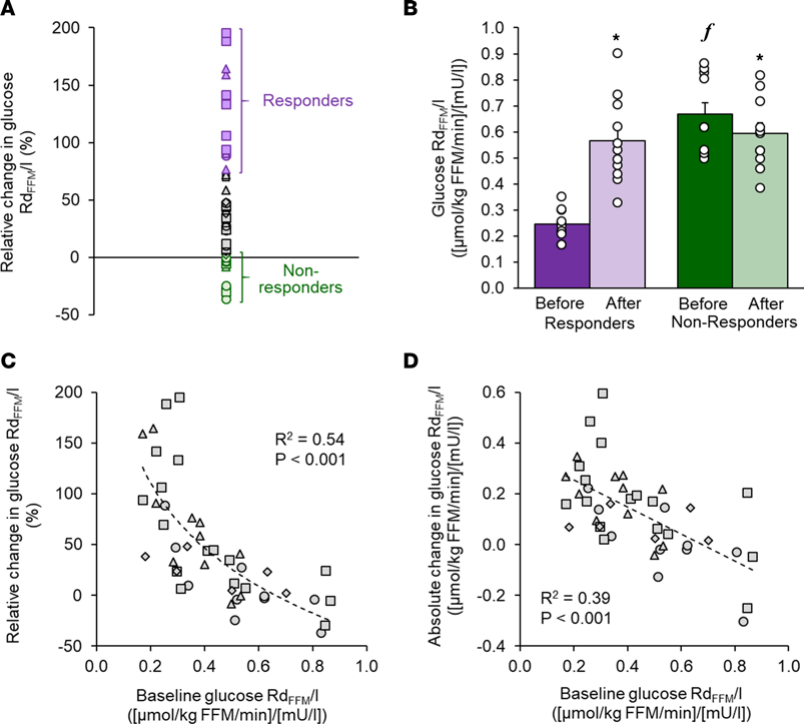
Glucose disposal rate during the hyperinsulinemic-euglycemic clamp procedure. (**A**) Change in the glucose disposal rate during the hyperinsulinemic-euglycemic clamp procedure, which provides an index of skeletal muscle insulin sensitivity, in 43 women with obesity after, compared with before, approximately 20% weight loss induced by a low-calorie diet (circles), laparoscopic adjustable gastric banding (triangles), Roux-en-Y gastric bypass (squares), or sleeve gastrectomy (diamonds). Purple symbols indicate Responders (top quartile values), green symbols indicate Non-responders (bottom quartile values). (**B**) Glucose disposal rate during the hyperinsulinemic-euglycemic clamp procedure before and after approximately 20% weight loss in Responders (*n* = 11) and Non-responders (*n* = 11). Data are mean ± SEM. *P < 0.05 versus corresponding value before weight loss. *^f^P* < 0.05 versus corresponding value in Responders. (**C** and **D**) Relationships between glucose disposal rate during the hyperinsulinemic-euglycemic clamp procedure at baseline (before weight loss) and the relative (**C**) and absolute (**D**) changes in glucose disposal rate after, compared with before, approximately 20% weight loss induced by a low-calorie diet (circles), laparoscopic adjustable gastric banding (triangles), Roux-en-Y gastric bypass (squares), or sleeve gastrectomy (diamonds) in 43 women with obesity. Repeated measures ANOVA was used to evaluate the effects of group and time on the outcome value in panel **B**. Regression analysis was used to evaluate the relationship between two variables in panels **C** and **D**. Rd_FFM_/I, glucose disposal rate, expressed per kg fat-free mass, in relation to plasma insulin concentration during the hyperinsulinemic-euglycemic clamp procedure.

The effect of marked (16%–25%) weight loss on insulin-stimulated glucose disposal rate in relation to FFM during the hyperinsulinemic-euglycemic clamp procedure, which provides an index of skeletal muscle insulin sensitivity (13), was used to identify Responders and Non-responders among 43 women with class II and class III obesity (body mass index ≥35 kg/m^2^). Plasma glucose and insulin concentrations during the clamp were not different before and after weight loss (glucose: 100 ± 0.5 and 101 ± 0.5 mg/dL, respectively; insulin: 119 ± 3 and 118 ± 3.7 mU/L, respectively). The relative change in insulin-stimulated glucose disposal rate after weight loss compared with values before weight loss ranged from –36% to 195%, with a median change of 33%. Responders were defined as participants in the highest quartile of change in insulin-mediated glucose disposal rate, which represented those with a greater than 75% increase (*n* = 11), whereas Non-responders were defined as those in the lowest quartile of change in insulin-mediated glucose disposal rate, which represented those with a less than 5% increase (*n* = 11) (Figure 1A).

Insulin-stimulated glucose disposal rate at baseline was approximately 60% lower in Responders than Non-responders (Figure 1B). Weight loss caused an approximately 75%–200% increase in insulin-stimulated glucose disposal rate in Responders but did not change or even decreased insulin-stimulated glucose disposal rate in Non-responders, so that the glucose disposal rate values after weight loss were not different between the 2 groups (Figure 1B). In the entire cohort of 43 women, both the absolute and the relative changes in insulin-stimulated glucose disposal rate induced by weight loss were negatively correlated with the insulin-stimulated glucose disposal rate at baseline (before weight loss) (Figure 1, C and D).

#### Liver insulin sensitivity.

**Figure 2:**
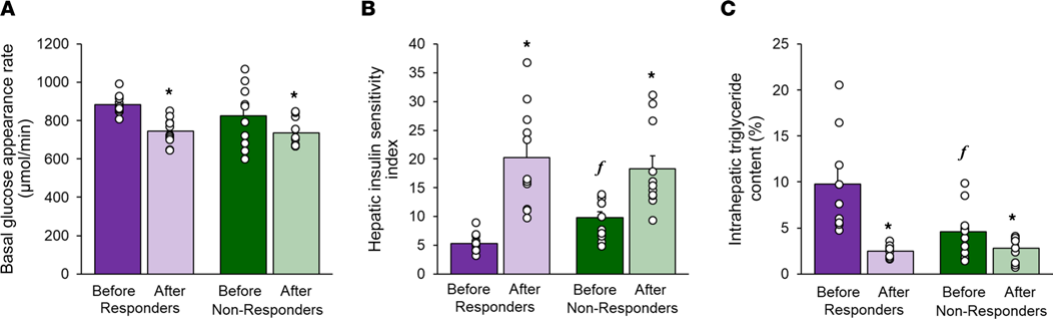
Basal hepatic glucose production rate, hepatic insulin sensitivity, and intrahepatic triglyceride content. (**A**–**C**) Basal glucose appearance rate in the circulation, which provides an index of hepatic glucose production rate (**A**), the hepatic insulin sensitivity index, which provides an assessment of insulin action on hepatic glucose production (**B**), and intrahepatic triglyceride content (**C**) before and after weight loss in Responders and Non-responders. Data are mean ± SEM. Repeated measures ANOVA was used to evaluate the effects of group and time on the outcome values. *P < 0.05 versus corresponding value before weight loss. *^f^P* < 0.05 versus corresponding value in Responders.

Basal glucose appearance rate in plasma, which primarily represents hepatic glucose production, and plasma glucose concentration were not different between Responders and Non-responders at baseline (Table 1 and Figure 2). However, plasma insulin concentration was twice as high in Responders compared with Non-responders (Table 1). Weight loss caused a decrease in basal glucose production rate and plasma glucose concentration in both groups without a difference between groups (Table 1 and Figure 2). Weight loss also caused a decrease in basal plasma insulin concentration in both groups, but the decrease in insulin was greater in Responders than Non-responders (Table 1 and Figure 2). The hepatic insulin sensitivity index was approximately 40% lower in Responders than Non-responders at baseline (Figure 2). Weight loss increased hepatic insulin sensitivity in both groups, but the increase was greater in Responders than Non-responders and the hepatic insulin sensitivity values after weight loss were not different between the 2 groups (Figure 2).

### Body composition

**Figure 3:**
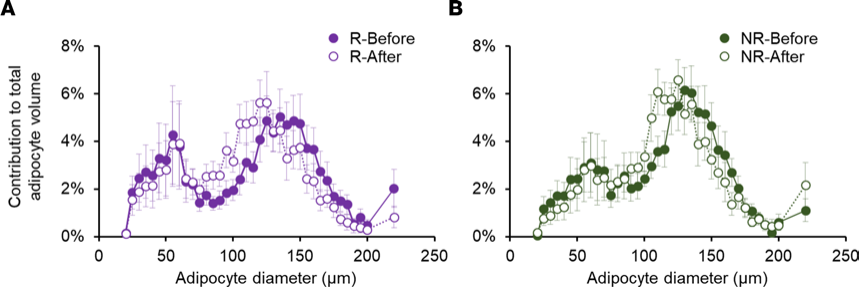
Subcutaneous abdominal adipocyte size. (**A** and **B**) Adipocyte size distribution profile in subcutaneous abdominal adipose tissue before and after weight loss in Responders (**A**) and Non-responders (**B**). Values are mean ± SEM. NR, Non-responders (*n* = 11); R, Responders (*n* = 9).

Before weight loss, percentage body weight as fat and the contribution of intra-abdominal adipose tissue volume to total abdominal (sum of intra-abdominal and subcutaneous abdominal) adipose tissue volume were not different between Responders and Non-responders, but intrahepatic triglyceride content was greater in Responders than Non-responders (Table 1 and Figure 2). Both Responders and Non-responders lost approximately 20% of their body weight (20.7% ± 2.8% and 19.2% ± 1.6%, respectively). The median amount of time and the range in the duration of time until target weight loss was achieved were not different between Responders (median: 21 weeks, range: 13–48 weeks) and Non-responders (median: 24 weeks, range: 16–67 weeks); the average rate of weight loss was 1.1% ± 0.1% per week in Responders and 0.8% ± 0.1% per week in Non-responders (*P* = NS). Although the decreases in total body fat and intra-abdominal adipose tissue volume were not different between groups, the absolute and relative decreases in intrahepatic triglyceride content were greater in Responders than Non-responders, so the intrahepatic triglyceride content after weight loss was not different between the 2 groups (Table 1 and Figure 2). The distribution of differently sized adipocytes in subcutaneous abdominal adipose was not different between Responders and Non-responders at baseline, and weight loss caused a shift toward smaller adipocytes in both Responders and Non-responders without a difference between groups (Figure 3).

### Plasma adipokines, markers of inflammation, and metabolites

At baseline, plasma leptin concentration was not different between groups, but high-molecular-weight adiponectin was lower in Responders than Non-responders (Table 1). Weight loss caused a decrease in plasma leptin and an increase in plasma adiponectin in both groups, and a decrease in the leptin/adiponectin concentration ratio that was greater in Responders than Non-responders (Table 1). Plasma plasminogen activator inhibitor 1 (PAI-1) concentration was higher in Responders than Non-responders at baseline and decreased after weight loss in both groups, but the decrease was greater in Responders than Non-responders (Table 1). Plasma C-reactive protein concentration decreased after weight loss in both Responders and Non-responders without a significant difference between groups (Table 1), and plasma interleukin 6 (IL-6), interferon γ (IFN-γ), monocyte chemoattractant protein 1 (MCP-1), tumor necrosis factor α (TNF-α), and RANTES (C-C motif ligand 5, CCL5) concentrations did not change after weight loss in either group (Table 1).

**Figure 4:**
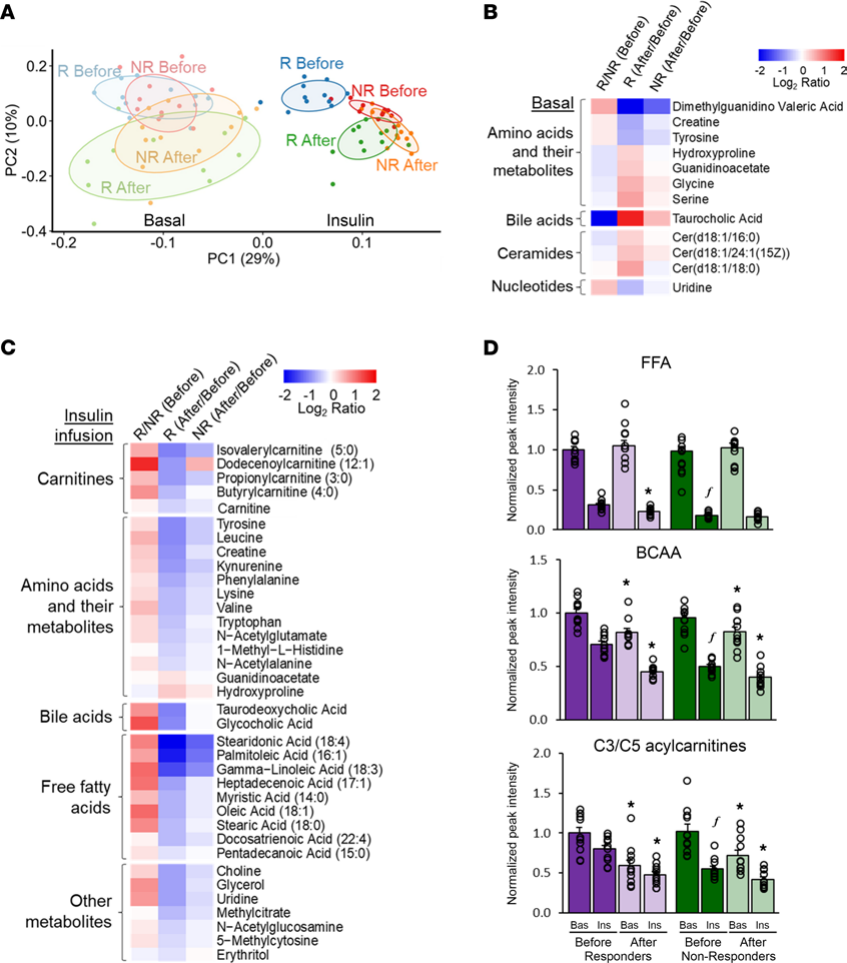
Plasma metabolite profile. (**A**) Principal component analysis of the 127 monitored plasma metabolites, shown with 68% confidence ellipses, in Responders (*n* = 11) and Non-responders (*n* = 11). (**B**) Baseline between group differences (R/NR Before) and weight-loss induced within group differences (After/Before) in the basal abundances of 12 metabolites for which repeated measures analysis of variance with group and time as factors revealed both a group × time interaction (*P* < 0.10) and a significant change (5% false discovery rate) with weight loss in Responders (*n* = 11) or both Responders and Non-responders (*n* = 11). (**C**) Baseline between group differences (R/NR Before) and weight-loss induced within group differences (After/Before) in the abundances of 36 metabolites during insulin infusion for which repeated measures ANOVA with group and time as factors revealed both a group × time interaction (*P* < 0.10) and a significant change (5% false discovery rate) with weight loss in Responders (*n* = 11) only or both Responders and Non-responders (*n* = 11). (**D**) Abundances of a priori–selected metabolites during basal conditions and during insulin infusion before and after weight loss in Responders (*n* = 11) and Non-responders (*n* = 11). Data are normalized to the Responders before weight loss value as reference. Data are mean ± SEM. Repeated measures ANOVA was used to evaluate the effects of group and time on the outcome values in panels **B**–**D**. *P < 0.05 versus corresponding value before weight loss. *^f^P* < 0.05 versus corresponding value in Responders. There was a significant (*P* < 0.05) effect of insulin (vs. basal) for free fatty acids, branched-chain amino acids, and acylcarnitines. BCAA, branched-chain amino acids; FFA, free fatty acids (sum of palmitate, oleate, and linoleate); NR, Non-responders; PC, principal component; R, Responders.

Principal component analysis of 127 annotated metabolites was used to evaluate the effect of weight loss and insulin infusion on the global plasma metabolite profile (Figure 4A). There was considerable overlap in the plasma metabolite profiles of Responders and Non-responders during basal conditions, both before and after weight loss. Insulin infusion caused a marked change in metabolite abundances in both groups before and after weight loss. Plasma metabolite abundances during insulin infusion were distinctly different between Responders and Non-responders before weight loss, but converged closer to each other with considerable overlap after weight loss because of a greater change in Responders than Non-responders (Figure 4A). Twelve of the 127 annotated metabolites assessed during basal conditions were affected differently by weight loss in Responders and Non-responders (Figure 4B). Dimethylguanidino valeric acid, creatine, tyrosine, and uridine decreased more and hydroxyproline, guanidinoacetate, glycine, serine, taurocholic acid, and 3 ceramide species increased to a greater extent in Responders than Non-responders (Figure 4B). During insulin infusion, weight loss caused a greater decrease in 34 metabolites (primarily acylcarnitines, amino acids, some bile acids, fatty acids, and glycerol) and a greater increase in 2 metabolites (guanidinoacetate and hydroxyproline) in Responders than Non-responders (Figure 4C).

We also evaluated the plasma concentrations of selected metabolites that have been implicated in the pathogenesis of insulin resistance, namely free fatty acids (FFAs) (14, 15), branched-chain amino acids (BCAAs) (16), and C3 and C5 acylcarnitines (17). The basal concentrations of these metabolites were not different between Responders and Non-responders before weight loss and did not change (FFAs) or decreased (BCAAs and C3/C5 acylcarnitines) after weight loss in both Responders and Non-responders, without differences between the 2 groups (Figure 4D). Insulin infusion decreased the plasma concentrations of these metabolites in both Responders and Non-responders before and after weight loss. However, plasma concentrations during insulin infusion before weight loss were higher in Responders than Non-responders and decreased more after weight loss in Responders than Non-responders, so that values during insulin infusion after weight loss were not different between groups (Figure 4D).

## Discussion

The ability of insulin to stimulate the disposal of circulating glucose, which primarily occurs in skeletal muscle and therefore represents skeletal muscle insulin sensitivity (13), is a key indicator of metabolic health. Our data demonstrate that there is considerable heterogeneity in the therapeutic effect of the same amount of marked (~20%) weight loss on muscle insulin action that is determined by baseline muscle insulin sensitivity; the improvement in insulin sensitivity induced by weight loss was negatively correlated with insulin sensitivity before weight loss. In fact, although weight loss in people with obesity typically increases insulin sensitivity (5, 6), we identified a subgroup of people in whom marked weight loss did not improve insulin-mediated glucose disposal. Weight loss improved hepatic insulin sensitivity, assessed by using the hepatic insulin sensitivity index, in both Responders (>75% increase in insulin-stimulated glucose disposal rate) and Non-responders, but the improvement was greater in Responders. In addition, weight loss caused a greater decrease in intrahepatic triglyceride content and greater changes in the plasma leptin/adiponectin ratio, plasma metabolites, and PAI-1 concentrations that are associated with insulin resistance. Marked weight loss also decreased several factors that have been implicated in causing insulin resistance, such as total body fat mass, intra-abdominal adipose tissue volume, adipocyte size, and circulating inflammatory markers, in both Responders and Non-responders, without differences between groups. These results demonstrate that the effect of marked weight loss on metabolic function is heterogeneous and muscle insulin action (insulin-stimulated glucose disposal) does not improve in people with obesity who already have “normal” muscle insulin sensitivity. However, marked weight loss still has therapeutic effects on other risk factors for cardiometabolic diseases in Non-responders.

Our study demonstrates that baseline muscle insulin sensitivity determines the improvement in muscle insulin sensitivity after marked weight loss. At baseline, insulin-stimulated glucose disposal was much lower in Responders than Non-responders, but increased to the same value observed in Non-responders after weight loss. Baseline insulin-stimulated glucose disposal, and additional markers of metabolic health associated with insulin action (hepatic insulin sensitivity index, plasma glucose and insulin concentrations, and intrahepatic triglyceride content; refs. (18, 19) in Non-responders were similar to values we have previously found in people who were lean and healthy and those with metabolically healthy obesity (18–23). These results are consistent with, and expand, the findings from previous studies that showed the improvement in whole-body insulin sensitivity, assessed by using an oral glucose tolerance test, insulin suppression test, or the glucose infusion rate during a hyperinsulinemic-euglycemic clamp procedure, after moderate (6%–14%) weight loss was blunted in people with obesity who were “insulin-sensitive” compared with those who were “insulin-resistant” (24–28). Together, the results from these studies and ours indicate the therapeutic benefit of moderate and even marked weight loss on multiorgan insulin sensitivity is attenuated in people who are already insulin sensitive and suggest there is a ceiling in the therapeutic effect of weight loss that is reached when values observed in people who are lean and healthy are achieved.

Our study is unable to determine the mechanism(s) responsible for the different effects of weight loss on multiorgan system insulin action in Responders and Non-responders. Nonetheless, our data demonstrate that total body and intra-abdominal adipose tissue volumes, subcutaneous adipocyte size, and many circulating inflammatory markers (CRP, MCP-1, CCL5, IL-6, IFN-γ, and TNF-α) are not involved, because there were no differences in these outcomes between groups. However, weight loss was associated with greater decreases in plasma PAI-1 concentration and in the plasma leptin/adiponectin concentration ratio in Responders than Non-responders. Increased plasma PAI-1 is associated with insulin resistance in people (22), and has been shown to cause insulin resistance in rodent models (29–31), and the plasma leptin/adiponectin concentration ratio correlates with insulin resistant glucose metabolism in people (32). Nonetheless, these data cannot determine whether the differences in PAI-1 and in the plasma leptin/adiponectin concentration ratio between Responders and Non-responders were a cause or an effect of the different changes in insulin sensitivity.

Plasma FFAs, BCAAs, and C3/C5 acylcarnitines have been implicated in causing insulin resistance (14–17, 33, 34), but their plasma concentrations are also regulated by insulin (17, 20, 35, 36). At baseline, basal plasma FFA, BCAA, and C3/C5 acylcarnitine concentrations were not different between Responders and Non-responders, but their concentrations were higher in Responders than Non-responders during insulin infusion. It is likely that the higher basal plasma insulin concentrations in Responders than Non-responders compensated for the insulin resistance in Responders, resulting in the same basal metabolite concentrations in both groups. However, the suppression of metabolite concentrations during insulin infusion (when insulin concentrations were the same in both groups) was blunted in Responders compared with Non-responders, demonstrating insulin resistance to their metabolic regulation in Responders. There was no differences in the effect of weight loss on basal FFA, BCAA, and C3/C5 acylcarnitine concentrations between Responders and Non-responders. However, weight loss caused a greater decrease in these metabolites during insulin infusion in Responders than Non-Responders, so plasma concentrations after weight loss were not different between groups. These results suggest that at baseline the metabolic pathways that regulate plasma concentrations of FFAs, BCAAs, and C3/C5 acylcarnitines (i.e., release into and removal from the systemic circulation) were more resistant to insulin in Responders than Non-responders. Weight loss caused a greater improvement in insulin sensitivity of these pathways in Responders than Non-responders, so that plasma concentrations during both basal conditions and during insulin infusion were not different between groups.

We conducted principal component and untargeted analyses to evaluate global differences in the plasma metabolome between Responders and Non-responders and to identify specific metabolites (in addition to the ones selected a priori) that differ between Responders and Non-responders. Our data demonstrate (i) a greater effect of weight loss on overall plasma metabolite abundances in Responders than Non-responders during both basal conditions and insulin infusion, and (ii) that although the plasma metabolome of Responders and Non-responders during the hyperinsulinemic clamp separated into distinct clusters, there was considerable overlap in circulating metabolite concentrations of the 2 groups after, but not before, weight loss. Our analyses also identified several specific plasma metabolites that changed more with weight loss during basal conditions in Responders than Non-responders, including greater increases in basal glycine, serine, guanidinoacetate, and hydroxyproline and greater decreases in dimethylguanidino valeric acid, creatine, tyrosine, and uridine in Responders than Non-responders. These metabolites are involved in 1-carbon metabolism, which supports the biosynthesis of nucleobases and epigenetic maintenance of physiological processes (37). In addition, glycine and serine, which increased more in Responders than Non-responders, are associated with increased insulin sensitivity, and dimethylguanidino valeric acid, tyrosine, and uridine, which decreased more in Responders than Non-responders, are associated with insulin resistance, hepatic steatosis, and increased risk of developing type 2 diabetes (38–43).

Our study has several limitations. First, different treatment approaches were used to achieve weight loss in our study participants, which could have influenced who was a Responder and Non-responder. However, the different weight loss interventions, namely a low-calorie diet only, laparoscopic adjustable gastric banding, sleeve gastrectomy, and Roux-en-Y gastric bypass, occurred in both Responders and Non-responders. Second, our study was focused on the effect of weight loss on insulin-resistant glucose metabolism in muscle and liver, and did not assess the effects on other obesity-associated comorbidities. Therefore, we cannot determine whether there would still be differences in the effect of weight loss between Responders and Non-responders on other obesity-related medical complications, such as obstructive sleep apnea, arthritis, and risk of cancer.

In conclusion, the present study demonstrates considerable heterogeneity in the therapeutic effect of marked (~20%) weight loss in people with obesity on insulin sensitivity, which was negatively correlated with baseline insulin sensitivity. Moreover, we identified a subgroup of participants in whom marked weight loss did not increase muscle insulin sensitivity at all, suggesting there is a ceiling in insulin-stimulated glucose disposal in those who are already insulin sensitive. These results have important implications in the medical management of people with obesity and support the need for developing personalized therapeutic goals that focus on clinical outcomes rather than weight loss alone.

## Methods

A total of 43 women with obesity (body mass index 44.1 ± 7.9 kg/m^2^) who participated in previously published (6, 11, 12) and ongoing studies that evaluated the effect of marked (16%–25%) weight loss on insulin-mediated glucose disposal by using the hyperinsulinemic-euglycemic clamp procedure in conjunction with stable isotopically labeled glucose tracer infusion were included in this study. All participants completed a comprehensive medical screening, including a medical history, physical examination, and standard blood tests before baseline testing; no participants had diabetes or were taking medications that affect insulin action. The relative change in insulin-stimulated glucose disposal rate induced by weight loss was used to identify Responders (highest quartile [>75% increase in insulin-mediated glucose disposal rate], *n* = 11) and Non-responders (lowest quartile [<5% increase in insulin-mediated glucose disposal rate], *n* = 11) (Figure 1). Five Responders and 8 Non-responders lost weight with diet therapy alone or laparoscopic adjustable gastric banding, whereas 6 Responders and 3 Non-responders lost weight after bariatric surgery (Roux-en-Y gastric bypass or sleeve gastrectomy). Eight of the 11 Responders were White, 2 were African American, and 1 was Native American. Five of the 11 Non-responders were White, 5 were African American, and 1 was Native American.

### Body composition.

Body fat mass and FFM were determined by using dual-energy x-ray absorptiometry. Intra-abdominal adipose tissue volume and intrahepatic triglyceride content were determined by using magnetic resonance imaging and spectroscopy, respectively.

### Hyperinsulinemic-euglycemic clamp procedure and adipose tissue biopsies.

Participants were admitted to the Clinical and Translational Research Unit at Washington University on the evening before the hyperinsulinemic-euglycemic clamp procedure and consumed a standard meal. At 0500 hours the following morning, after participants fasted for 10 hours overnight, one catheter was inserted into a forearm vein to infuse a stable isotopically labeled glucose tracer (Cambridge Isotope Laboratories), dextrose, and insulin; a second catheter was inserted into a radial artery to obtain blood samples. If radial artery cannulation was not possible, a catheter was inserted into a contralateral hand vein, which was heated to 55°C by using a thermostatically controlled box, to obtain arterialized blood samples. At approximately 0600 hours, a primed, continuous infusion of [6,6-^2^H_2_]glucose was started and maintained for 3.5 hours. The hyperinsulinemic clamp was initiated with a 2-step priming dose of 200 and 100 mU insulin per m^2^ body surface area per minute for 5 minutes each, and then maintained with an insulin infusion rate of 50 mU/m^2^ body surface area per minute for 3.5 hours. Euglycemia (~100 mg/dL) during insulin infusion was maintained by infusing 20% dextrose containing 2.5% [6,6-^2^H_2_]glucose. Blood samples to determine glucose kinetics and plasma metabolite and hormone concentrations were collected during the last 30 minutes of the basal period and the insulin infusion, respectively (6, 11); a subcutaneous abdominal adipose tissue biopsy sample was obtained by using the miniliposuction technique during the basal period to determine adipocyte size by using an automated Coulter counter (44).

### Weight loss intervention and repeat testing.

After baseline testing was completed, participants participated in a supervised diet and behavioral therapy weight loss program (*n* = 11) or had bariatric surgery (laparoscopic adjustable gastric banding, Roux-en-Y gastric bypass, or sleeve gastrectomy; *n* = 32). After 16%–25% weight loss was achieved, participants were prescribed a weight-maintenance diet in an effort to maintain a stable body weight (≤2% change) for at least 3 weeks before the body composition analyses and hyperinsulinemic-euglycemic clamp and adipose tissue biopsy procedures were repeated.

### Plasma hormones and markers of inflammation.

Insulin concentration was measured by using an electrochemiluminescent immunoassay (Elecsys; Roche Diagnostics). Leptin concentration was determined by using a radioimmunoassay (Merck Millipore). High-molecular-weight adiponectin concentration was measured by using an ELISA kit (R&D Systems). Plasma concentrations of PAI-1, MCP-1, RANTES/CCL5, IL-6, IFN-γ, and TNF-α were determined on a Luminex platform with multiplex kits (R&D Systems).

### Plasma metabolites.

Plasma metabolite ion abundances were determined by using multiple liquid chromatography–tandem mass spectrometry (LC-MS/MS; Thermo Vanquish UPLC and Thermo QExactive orbitrap MS) methods, as previously described (45). Polar metabolites were assessed by chromatographic separation of deproteinized plasma on a Millipore SeQuant Zic-pHILIC column before MS/MS analysis; the mzMine 2.30 software suite (http://mzmine.github.io/download.html) was used for data processing. Lipids were determined by passing deproteinized plasma through a Phenomenex Strata-X solid phase extraction plate before LC-MS/MS analysis; Progenesis QI software (Nonlinear Dynamics) was used for data processing. Ceramides and dihydroceramides were chromatographically separated on an Agilent Eclipse Plus RRHD C18 column before LC-MS/MS analysis; mzMine 2.30 was used for data processing. Our analysis focused on 127 annotated metabolites that were identified by matching their mass, retention times, and MS/MS patterns to an in-house library of commercial standards.

Principal component analysis was used to identify potential differences in the global plasma metabolite profile among Responders and Non-responders before and after weight loss and during basal conditions and insulin infusion. Between- and within-group differences in the 127 metabolites and a set of a priori–selected metabolites and metabolite classes that are associated with insulin resistance — FFAs (14, 15), BCAAs (16, 33), and C3/C5 acylcarnitines (17) — were analyzed by using ANOVA with time (before and after weight loss) as within-subjects factor and group (Responder vs. Non-responder) as between-subjects factor. When a significant group × time interaction was identified, paired and unpaired 2-tailed Student’s *t* tests were used as needed to test for statistical differences between groups at baseline and within-group changes induced by weight loss; *P* values from these post hoc analyses on the global profile (127 metabolites) were adjusted to a <5% false discovery rate (Benjamini-Hochberg method).

### Calculations.

Glucose rate of appearance (Ra) in plasma was calculated by dividing the glucose tracer infusion rate by the average plasma glucose tracer-to-tracee ratio during the last 30 minutes of the basal period and during the last 30 minutes of the hyperinsulinemic-euglycemic clamp procedure. Glucose Ra during basal conditions represents endogenous glucose Ra, an index of hepatic glucose production rate, which equals basal glucose disposal rate (Rd) from plasma. Hepatic insulin sensitivity was determined as the reciprocal of the product of the basal glucose Ra (in μmol/min) and fasting plasma insulin concentration (in mU/L × 10^5^; ref. 46). Glucose Rd during the clamp procedure, an index of insulin-mediated tissue glucose uptake (13), was calculated as total glucose Ra (i.e., the sum of endogenous glucose Ra and the rate of infused glucose). Insulin sensitivity of tissue glucose uptake was determined as glucose Rd relative to FFM during insulin infusion divided by the plasma insulin concentration during the clamp procedure.

### Statistics.

Paired, 2-tailed Student’s *t* test was used to evaluate the effect of weight loss on body composition, basic metabolic characteristics, and insulin sensitivity at baseline. The response to weight loss in Responders and Non-responders was compared by using ANOVA. A *P* value of 0.05 or less was considered statistically significant. Data are presented as mean ± SD unless otherwise indicated. Statistical analyses were performed by using SPSS Statistics v26 (IBM).

### Study approval.

All participants provided written informed consent before participating in the research protocols, which were approved by the Institutional Review Board of Washington University in St. Louis, Missouri.

## Author contributions

BM and SK designed the study. MY, JY, BWP, and JCE assisted in conducting the clinical studies and sample processing. BDK, JDW, MJ, and BM conducted the metabolomics analyses. MY, BDK, BWP, JY, BM, and SK analyzed and interpreted the data and wrote the manuscript. All authors critically reviewed and edited the manuscript.

## Supplementary Material

ICMJE disclosure forms

## Figures and Tables

**Table 1:**
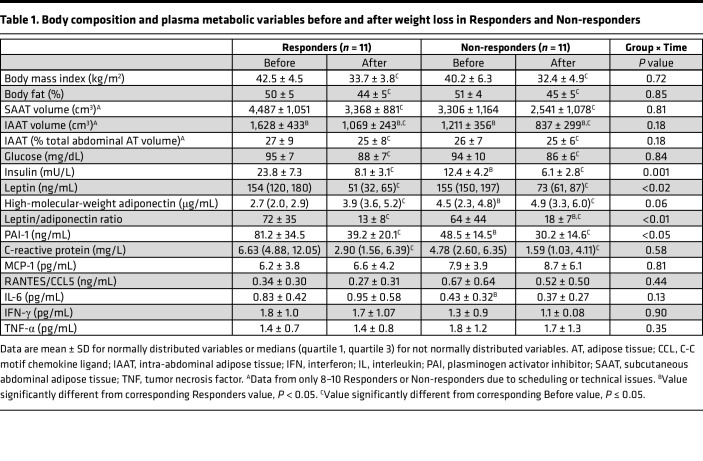
Body composition and plasma metabolic variables before and after weight loss in Responders and Non-responders

## Version 1 05/09/2023

In-Press Preview

## Version 2 06/22/2023

Electronic publication
